# Comparative analysis of seed and seedling irradiation with gamma rays and carbon ions for mutation induction in Arabidopsis

**DOI:** 10.3389/fpls.2023.1149083

**Published:** 2023-04-06

**Authors:** Yoshihiro Hase, Katsuya Satoh, Satoshi Kitamura

**Affiliations:** Takasaki Advanced Radiation Research Institute, National Institutes for Quantum Science and Technology (QST), Takasaki, Gunma, Japan

**Keywords:** mutation, carbon ion beam, gamma ray, Arabidopsis, structural variation

## Abstract

The molecular nature of mutations induced by ionizing radiation and chemical mutagens in plants is becoming clearer owing to the availability of high-throughput DNA sequencing technology. However, few studies have compared the induced mutations between different radiation qualities and between different irradiated materials with the same analysis method. To compare mutation induction between dry-seeds and seedlings irradiated with carbon ions and gamma rays in Arabidopsis, in this study we detected the mutations induced by seedling irradiation with gamma rays and analyzed the data together with data previously obtained for the other irradiation treatments. Mutation frequency at the equivalent dose for survival reduction was higher with gamma rays than with carbon ions, and was higher with dry-seed irradiation than with seedling irradiation. Carbon ions induced a higher frequency of deletions (2−99 bp) than gamma rays in the case of dry-seed irradiation, but this difference was less evident in the case of seedling irradiation. This result supported the inference that dry-seed irradiation under a lower water content more clearly reflects the difference in radiation quality. However, the ratio of rearrangements (inversions, translocations, and deletions larger than 100 bp), which are considered to be derived from the rejoining of two distantly located DNA breaks, was significantly higher with carbon ions than gamma rays irrespective of the irradiated material. This finding suggested that high-linear energy transfer radiation induced closely located DNA damage, irrespective of the water content of the material, that could lead to the generation of rearrangements. Taken together, the results provide an overall picture of radiation-induced mutation in Arabidopsis and will be useful for selection of a suitable radiation treatment for mutagenesis.

## 1 Introduction

Mutation breeding contributes greatly to the generation of new plant varieties and genetic resources. According to the Joint FAO/IAEA Mutant Variety Database, gamma rays are the most widely used mutagen in the world (Joint FAO/IAEA). However, accelerated ion beams, which deposit much higher energy along the path of ion particles, have attracted attention as a mutagen because the energy deposition profile is quite different from that of gamma rays (Goodhead, 1994; Blakely and Kronenberg, 1998). Early studies on ion-beam utilization suggested that ion beams have a broader mutation spectrum than gamma rays in practical mutation breeding, although the underlying differences in the induced mutations were unclear at that time (Okamura et al., 2003; Tanaka et al., 2010; Shu et al., 2012). In the following decade, the availability of high-throughput sequencing technology at relatively low cost has shed considerable light on the molecular nature of induced mutations at the genome-wide level (Hirano et al., 2015; Du et al., 2017; Kazama et al., 2017; Hase et al., 2018; Ichida et al., 2019; Jo and Kim, 2019; Li et al., 2019; Yang et al., 2019; Oono et al., 2020; Hase et al., 2020; Lee et al, 2021; Du et al., 2022; Kitamura et al., 2022). The effects of ion beams are reported to largely depend on the value of linear energy transfer (LET), which represents the amount of energy transferred to the irradiated material per unit length. The ion beams with extremely high LET (e.g., argon ions, 640 keV/µm) are suggested to often induce drastic and complex structural alterations of chromosomes, whereas ion beams with moderate LET (e.g., carbon ions, 30 keV/µm) are suggested to often induce relatively small insertion and deletion (InDel) mutations, which is beneficial to induce loss-of-function gene mutations (Hirano et al., 2015; Kazama et al., 2017). We consider that this knowledge will provide guidance in choosing a suitable mutagenic treatment to maximize the probability of obtaining a desired mutant with a reasonably achievable size of mutant population. In most previous studies described above, dry seeds were used as the irradiation material, whereas many types of plant tissues, such as seeds, seedlings, and lateral buds, are mutagenized in a practical mutation breeding program. In addition, water content is known to greatly affect the radiation sensitivity because it enhances an indirect radiation action by generating highly reactive free radicals (Yokoya et al., 2003; Yokoya et al., 2008; Vochita et al., 2014). However, to date, few studies have compared the induced mutations between different radiation qualities and also between different irradiated materials with the same analysis method.

In a previous study, we compared carbon-ion (107 keV/µm) induced mutations after dry-seed and seedling irradiation in Arabidopsis and showed that dry-seed irradiation resulted in a higher mutation frequency than did seedling irradiation at the same effective dose as judged by the survival rate (Hase et al., 2018). We also examined gamma-ray-induced mutations after dry-seed irradiation and showed that gamma rays induced a significantly higher number of total mutation events than carbon ions after dry-seed irradiation (Hase et al., 2020). Furthermore, we showed that gamma rays predominantly induced single-base substitutions (SBSs), whereas carbon ions frequently induced deletions ≥ 2 bp after dry-seed irradiation. In the present study, we examined gamma-ray-induced mutations after seedling irradiation, which has not previously been performed, and analyzed the data together with the previously obtained data to provide an overall picture of radiation-induced mutation in Arabidopsis.

Ionizing radiation induces various types of mutation: SBS, InDel, and structural variation (SV), such as inversion and translocation. In general, SVs are more difficult to detect by mutation detection algorithms from short-read sequencing data compared with the other types of mutation (Zheng et al., 2021). Given that the detection efficiency differs depending on the type of mutation and the type of algorithm, combined use of different algorithms is considered to be effective to achieve efficient and unbiased mutation detection (Kosugi et al., 2019). Four approaches – read pair (RP), read depth (RD), split read (SR), and assembly (AS) approaches – have mainly been used to detect SVs, and the recently developed algorithms mostly use a combination of approaches. Kosugi et al. (2019) comprehensively evaluated the performance of 69 algorithms and selected five (GRIDSS, Lumpy, SVseq2, SoftSV, and Manta) as superior algorithms for detecting SVs. In our previous research (Hase et al., 2018; Hase et al., 2020), we used the Pindel and BreakDancer algorithms to detect SVs, which use SR and RP alone, respectively. This raises a concern about SVs being underestimated compared with the other types of mutation. In the present study, Manta (which uses the RP, SR, and AS approaches) and Lumpy (RP, SR, and RD), were additionally used to detect mutations that were not detected using our previous method.

## 2 Materials and methods

### 2.1 Gamma irradiation and survival rate

Seeds of *Arabidopsis thaliana* (Columbia accession) obtained from a single plant were used to minimize the background mutations that existed in the laboratory strain. Dry seeds were sown in plastic dishes filled with a culture soil (TM-2, Takii & Co., Ltd., Kyoto, Japan), and appropriately watered with deionized water. The dishes were kept in a growth room (Koito Industries, Yokohama, Japan) at 23°C under a 16-h light/8-h dark photoperiod with ~70 µmol m^2^/s fluorescent light. One-week-old seedlings were exposed to ^60^Co gamma rays for 30 min with 50 to 200 Gy at the Takasaki Advanced Radiation Research Institute, National Institutes for Quantum Science and Technology. Irradiated seedlings were transplanted to plug trays (200 cells/tray, Takii & Co., Ltd.) filled with a 1:1 mixture of culture soil (TM-2) and vermiculite (medium size, Vern-piece; Hakugen Co., Ltd., Tokyo, Japan). The survival rate was determined 3 weeks after irradiation. Seedlings with more than five fresh and viable true leaves were counted as survivors. Three replications of 30 seedlings were used for each dose. Survival curves were generated on the basis of the single hit–multitarget theory using the following equation as previously described (Hase et al., 2012):


$$\mathrm{Survival rate}=1-(1-e^{-D/D_{0}}{)}^{m}\text{,}$$

where *D* (the dose), *D*_0_ (the dose conferring 37% survival), and *m* (the extrapolated number) are the parameters. The data were fitted using the least-squares method with KaleidaGraph (Synergy Software, Reading, PA, USA). The shoulder dose (*D*_q_) of the survival curves was calculated using the equation:


$$D\text{q}=D_{0}\times \text{ln}\;m$$

Seeds of the following generation (M_2_ lines) were collected from individual plants.

### 2.2 Whole-genome resequencing

Ten M_2_ lines were randomly chosen for 75 Gy and 125 Gy irradiation. Genomic DNA was isolated from a single randomly selected plant for each M_2_ line with the DNeasy Plant Mini Kit (QIAGEN K.K., Tokyo, Japan). Sequencing libraries were prepared using the KAPA HyperPlus Kit (Nippon Genetics Co., Ltd., Tokyo, Japan) and TruSeq DNA and RNA UD Indexes (Illumina K.K., Tokyo, Japan). The libraries were sequenced with an Illumina HiSeq X Ten platform to generate 150 bp paired-end reads. Low-quality reads were removed using Illumiprocessor (version 2.0.9; https://illumiprocessor.readthedocs.io/en/latest/). The clean reads were mapped to the Arabidopsis reference genome (TAIR10) using BWA (version 0.7.5; http://bio-bwa.sourceforge.net/), SAMtools (version 1.3.1; http://samtools.sourceforge.net/), and Picard-tools (version 1.119; https://broadinstitute.github.io/picard/). The candidate mutation sites were identified using the GATK Haplotype Caller (version 3.4; https://software.broadinstitute.org/gatk/), Pindel (version 0.2.4; http://gmt.genome.wustl.edu/packages/pindel/user-manual.html), and BreakDancer (version 1.4.5; http://breakdancer.sourceforge.net/) algorithms as previously described (Hase et al., 2020). The Manta (version 1.6.0; https://github.com/Illumina/manta) and Lumpy (version 0.3.1; https://github.com/arq5x/lumpy-sv) algorithms were used to further detect structural variants with the default settings. The candidate mutation sites detected by GATK in more than two independent samples were excluded as false positives. The candidate mutation sites with allele frequencies (AF; proportions of mutant reads at a site) ≤ 25% were also excluded. The mutation site was considered heterozygous if 25%< AF< 80% and AF< 5% for all other samples. The mutation site was considered homozygous if AF ≥ 80% and AF< 5% for all other samples. All candidate mutations were confirmed with Integrative Genomics Viewer (IGV; version 2.8.13; http://software.broadinstitute.org/software/igv/). The number of mutation sites during the filtering process after detection with GATK was shown in **Supplementary Table 1**. For mutation detection using Pindel, BreakDancer, Manta, and Lumpy, the candidate mutation sites unique to a single sample were selected and confirmed by IGV.

The identified mutations were classified into eight categories: (1) SBS; (2) single-base insertion (+1); (3) single-base deletion (−1); (4) insertion of 2−99 bp (Ins 2−99 bp); (5) deletion of 2−99 bp (Del 2−99 bp); (6) deletion of 100 bp or more (Del ≥ 100 bp); (7) SV; and (8) complex type. In the case that the InDels included insertion of unknown sequences, the length of the sequence alteration represented the difference from the reference sequence, e.g., a putative 10-base deletion accompanied by a 3-base insertion of unknown sequence was considered to be a 7-base deletion. The complex type represented more than two consecutive SBSs, or more than two SBSs and/or short InDels identified with a gap of less than 10 bases. In such cases, two or more bases identical to the reference genome were assumed to represent a non-mutated sequence. The SV category comprised inversions and translocations. Insertion of 100 bp or more was not detected in this study. Mutation frequency (MF) was calculated as the number of mutation events divided by the length of the reference genome.

## 3 Results

### 3.1 Radiation sensitivity of dry seeds and seedlings

**Figure 1:**
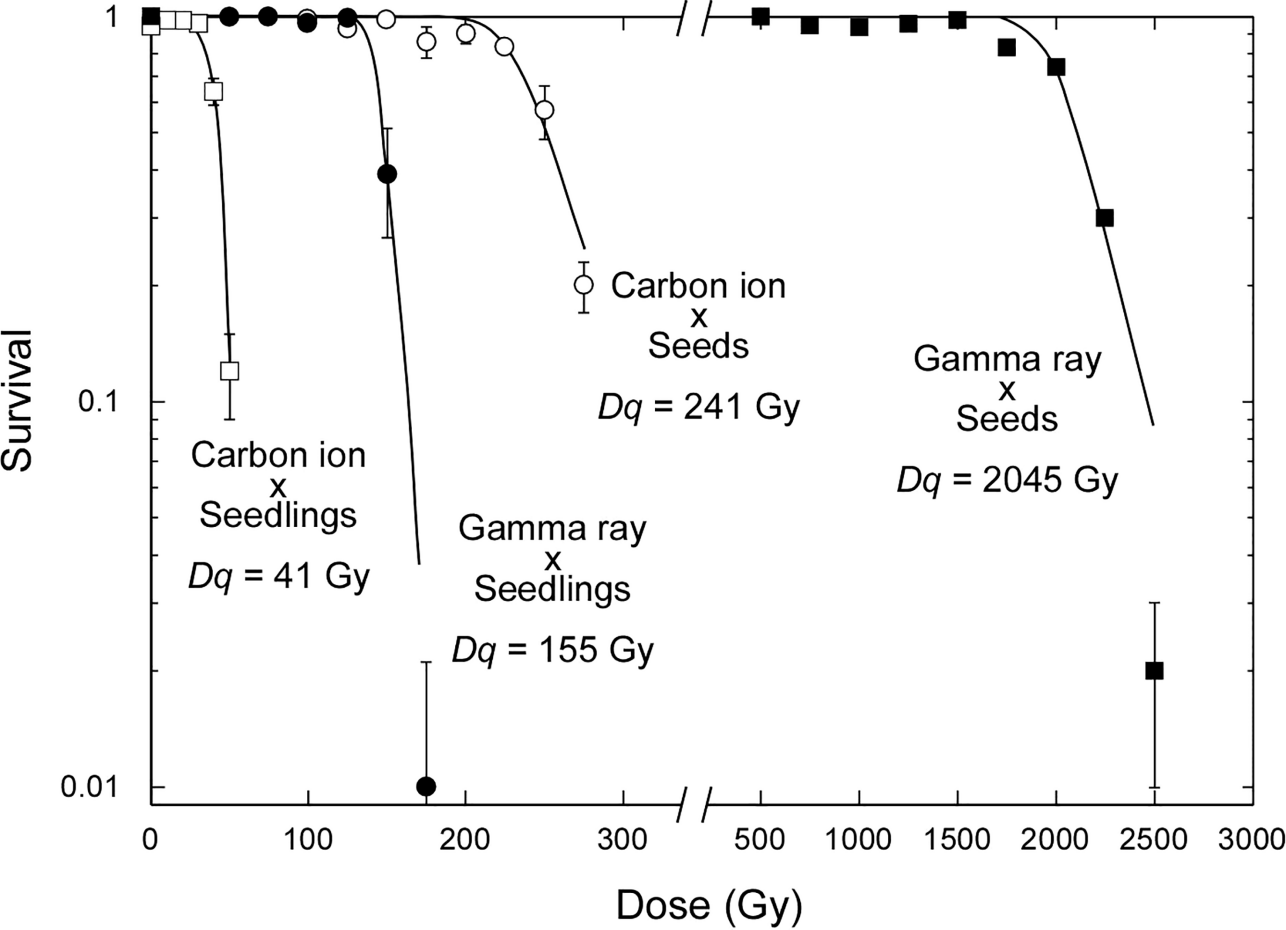
Dose–response relationships for survival rate of Arabidopsis dry seeds and 7-day-old seedlings irradiated with 17.3 MeV/u carbon ions or gamma rays. Data points are the mean ± standard error of three replications with more than 25 plants. Survival curves were drawn on the basis of the single hit–multitarget theory as previously described (Hase et al., 2012). *D*_q_ is the shoulder dose. Data for gamma irradiation of seedlings were newly obtained in the present study. All other data are from our previous studies (Hase et al., 2018; Hase et al., 2020).

It is well known that seedlings are much more sensitive to ionizing radiation than dry seeds. The water content is considered to be the most important factor. Interestingly, the sensitizing effect of the water content differed between carbon ions and gamma rays. Seedlings were 5.9 times more sensitive than dry seeds in the case of carbon ions, as judged by the doses corresponding to the shoulder dose of the survival curves (*D*_q_), whereas seedlings were 13.2 times more sensitive than dry seeds in the case of gamma rays (**Figure 1**). This difference reflects that carbon ions have a higher proportion of direct actions of accelerated charged particles, whereas gamma rays have a higher proportion of indirect actions *via* radiolysis of water molecules. The relative effectiveness of carbon ions compared with that of gamma rays per dose was 8.5 in the case of dry-seed irradiation and 5.9 in the case of seedling irradiation. This finding supports the inference that dry-seed irradiation under a lower water content more clearly reflects the difference in radiation quality.

### 3.2 Mutation detection from seedlings irradiated with gamma rays

Seven-day-old seedlings irradiated with 75 or 125 Gy of gamma rays, which corresponded to ~50% and ~75% of *D*_q_, respectively, were used for detection of mutations, as performed in the other material–radiation combinations (**Table 1**). Ten randomly chosen independent M_2_ plants for each dose were subjected to whole-genome resequencing. The mean depth of coverage was 41.4 and 99.8% of the target bases were covered at a minimum of 10× (**Supplementary Table 2**). In total, 822 mutation events were detected from the 20 samples using the GATK, Pindel, and BreakDancer algorithms. In our previous research (Hase et al., 2018; Hase et al., 2020), 418 mutation events from 12 M_2_ plants derived from dry-seed irradiation with carbon ions, 249 mutation events from 12 M_2_ plants derived from seedling irradiation with carbon ions, and 1023 mutation events from 14 M_2_ plants derived from dry-seed irradiation with gamma-rays were detected using the GATK, Pindel, and BreakDancer algorithms.

**Table 1:** Experimental conditions that we performed whole genome resequencing.

Material-radiation combination	*D_q_* (Gy)	Dose and the number of M_2_ plants	
		Dose 1 (~50% of *D_q_*)	Dose 2 (~75% of *D_q_*)
Gamma ray × dry seeds	2045	1000 Gy (49% of *D_q_*) 6 plants	1500 Gy (73% of *D_q_*) 8 plants
Gamma ray × seedlings	155	75 Gy (48% of *D_q_*) 10 plants	125 Gy (81% of *D_q_*) 10 plants
Carbon ion × dry seeds	241	125 Gy (52% of *D_q_*) 6 plants	175 Gy (73% of *D_q_*) 6 plants
Carbon ion × seedlings	41	20 Gy (49% of *D_q_*) 6 plants	30 Gy (73% of *D_q_*) 6 plants

*D*_q_ is the shoulder dose. Data for gamma irradiation of seedlings were newly obtained in the present study. All other data are from our previous studies (Hase et al., 2018; Hase et al., 2020).

### 3.3 Mutation detection using Manta and Lumpy algorithms

**Figure 2:**
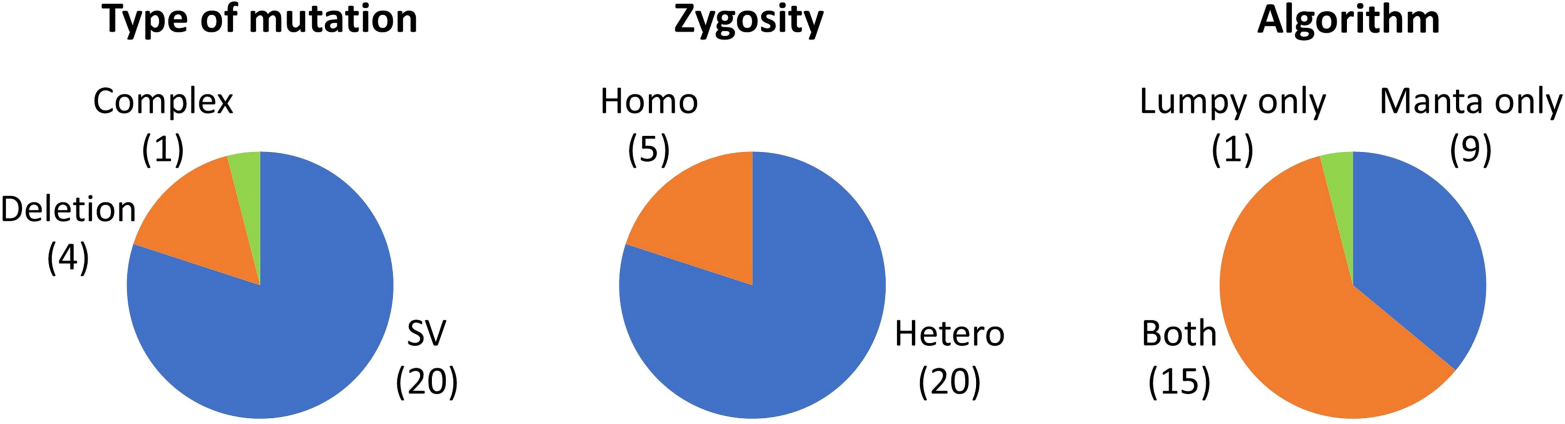
Mutations newly detected by the Manta and Lumpy algorithms. Numbers in parentheses indicate the number of mutations that correspond to each category.

We sought to detect undetected mutations, particularly SVs, using the Manta and Lumpy algorithms. The sequence data for a total of 58 samples shown in **Table 1** were reanalyzed and 25 mutation events were newly detected (**Supplementary Table 3**). As shown in **Figure 2**, 20 of the 25 mutation events were SVs and, in addition, four large deletions ranging from 25 to 380 kb and one complex-type mutation were detected. With regard to the zygosity, 20 of the 25 mutation events were heterozygous mutations. More than half of the mutations were detected by both algorithms, whereas Manta was more effective than Lumpy under our experimental conditions. Confirmation with IGV strongly supported the occurrence of structural alterations; however, the overall structure, particularly of heterozygous SVs, is sometimes difficult to deduce. Ultimately, we succeeded in deducing the overall structure for 12 of the 20 SV events. The sequencing reads located at the junction point reasonably connected with those at another junction point in most cases of simple inversion and reciprocal translocation, which have only two major junction points (**Supplementary Figures 1A, C, F**). However, we failed to deduce the overall structure when the sequencing reads at junction points connected with a highly repetitive region (**Supplementary Figure 1B**), or when the SV had three or more heterozygous junction points (**Supplementary Figures 1D, E**). The newly detected 25 mutations accounted for only 1% of the 2556 mutation events detected in the 58 samples; however, if only the SV events were considered, the number increased by 1.7 times from 30 to 50 events. These results demonstrated that the utilization of additional algorithms particularly enhanced the detection efficiency of heterozygous SV events.

### 3.4 MF and characteristics of mutations

**Figure 3:**
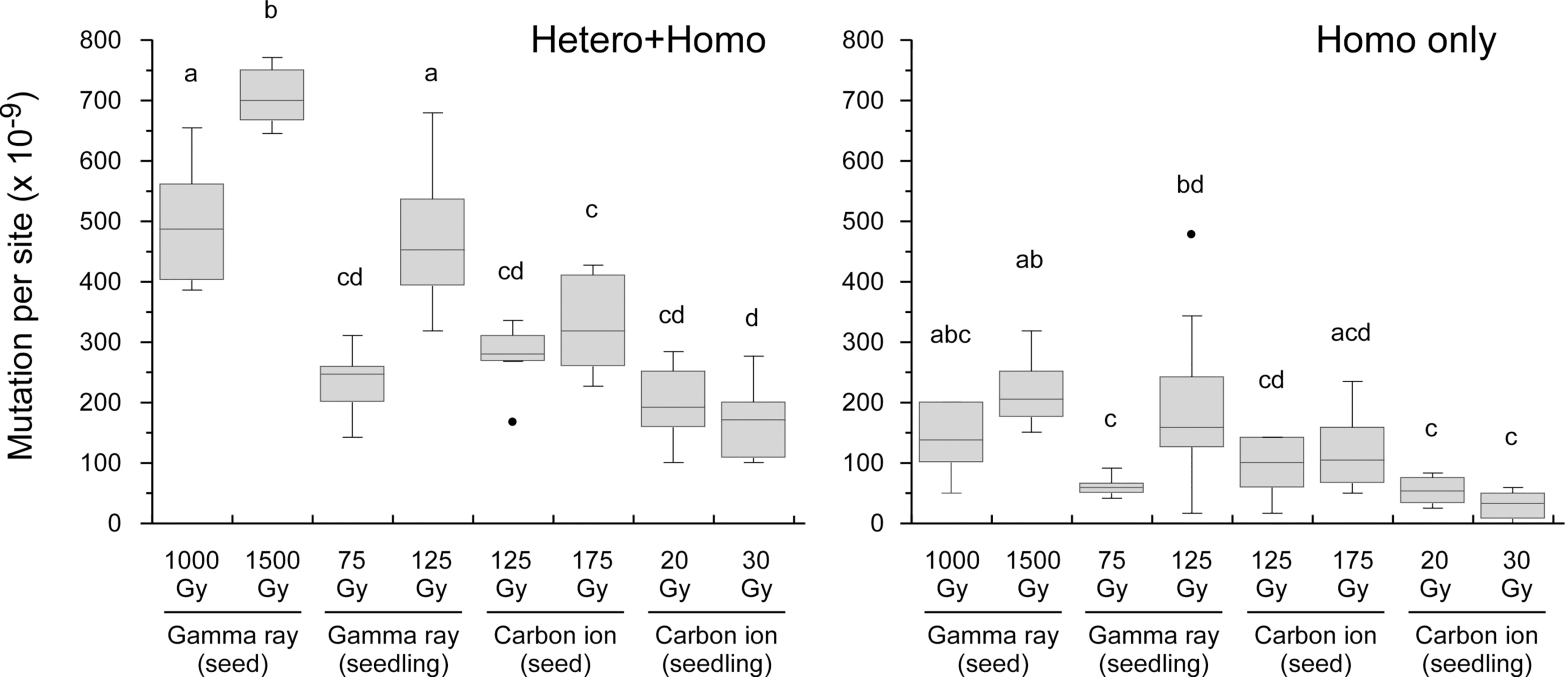
Mutation frequency in each treatment group shown in box and whisker plots. The box presents the first and third quartiles with the center line at the median, while whiskers extend to the minimum and maximum values. The dots present outliers. The right panel presents data for homozygous mutations only and the left panel presents data for both homozygous and heterozygous mutations. Different lowercase letters above boxes in each panel indicate a significant difference (one-way ANOVA with multiple comparison test, *p*< 0.05).

The MFs in all material–radiation combinations are shown in **Figure 3**. In general, the MF was higher in the order of (1) gamma ray > carbon ion and (2) dry-seed irradiation > seedling irradiation at the equivalent doses for survival reduction. The dose dependency between doses 1 and 2 (~50% and ~75% of *D*_q_ shown in **Table 1**) was observed in the case of gamma rays irrespective of the irradiated material; however, it was unclear in the case of carbon ions.

**Figure 4:**
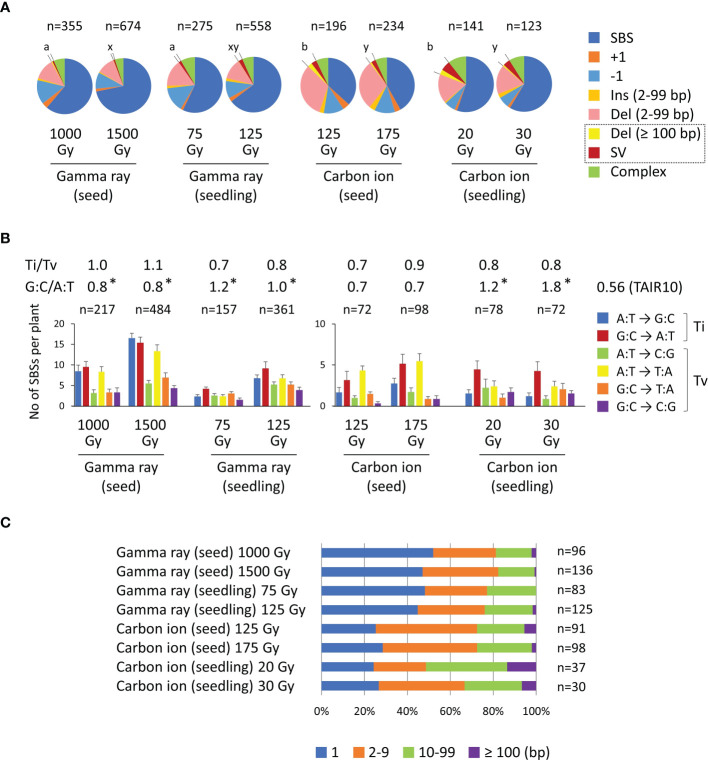
Characterization of mutations induced in Arabidopsis dry seeds and 7-day-old seedlings irradiated with 17.3 MeV/u carbon ions or gamma rays. The data in this figure include both homozygous and heterozygous mutations. **(A)** All mutation types detected in each treatment. The proportions of rearrangements (Deletion ≥ 100 bp and structural variation [SV]) are indicated by dotted lines. Statistical comparisons of the proportions of rearrangements were performed at the equivalent dose on survival reduction. Different lowercase letters indicate a significant difference (a and b are for a comparison at dose 1 (~50% of *D_q_*); x and y are for a comparison at dose 2 (~75% of *D_q_*); Fisher’s exact test with multiple comparison correction, *p*< 0.05). **(B)** Spectra of single-base substitutions. Complementary substitutions (e.g., G:C to A:T and C:G to T:A) were merged. The *T*_i_/*T*_v_ ratio represents the ratio of total transition to transversion events. The G:C/A:T ratio represents the ratio of G:C pairs to A:T pairs in the original nucleotides that underwent substitutions. Asterisks indicate significant differences from the A:T/G:C ratio (0.56) of the TAIR10 reference sequence (Fisher’s exact test, *p*< 0.05). **(C)** Distribution of deletion sizes.

In this study, we compared the characteristics of mutations irrespective of the zygosity otherwise indicated, because the ratio of homozygous to heterozygous mutations did not differ from the theoretically expected ratio of 0.5 in the M_2_ generation. Data only for homozygous mutation events are shown in **Supplementary Figure 2**. As previously reported (Hase et al., 2020), carbon ions induced Del (2−99 bp) mutations more frequently than did gamma rays in the case of dry-seed irradiation (shown in pink in **Figure 4A**). However, this characteristic was not evident in the case of seedling irradiation. This result was consistent with the inference that dry-seed irradiation with a lower water content more clearly reflected the difference in radiation quality.

The SV and Del ≥ 100 bp mutations were merged and categorized as rearrangements in previous studies, because both were considered to be derived from rejoining of two distantly located DNA double-strand breaks (DSBs) (Kazama et al., 2017; Li et al., 2019). The relevance of this classification was also supported by a similar feature observed at the rejoined sites of SV and Del ≥ 100 bp mutations with regard to the frequency of insertion and microhomology (Kitamura et al., 2022). Carbon ions showed a higher proportion of rearrangement events than gamma rays at the equivalent effective survival dose (**Figure 4A**). Approximately half of the M_2_ plants contained rearrangement events in the case of gamma rays, whereas approximately 80% of the M_2_ plants contained rearrangement events in the case of carbon ions (**Table 2**). Furthermore, carbon ions induced almost twice the number of rearrangement events per M_2_ plant than gamma rays. No significant difference was observed between dry-seed and seedling irradiation regarding the frequency of rearrangement events (**Figure 4A**).

**Table 2:** Frequency of occurrence of rearrangement events per plant.

	Gamma ray (dry seed)	Gamma ray (seedling)	Carbon ion (dry seed)	Carbon ion (seedling)
The percentage of plants that contain rearrangement events ^1^	57% (8/14)	45% (9/20)	83% (10/12)	75% (9/12)
The mean number of rearrangement events per plant ^2^	0.8 (11/14)	0.9 (18/20)	1.6 (19/12)	1.5 (18/12)

^1^ Numbers in parenthesis indicates total number of plants that contain at least one rearrangement event/total number of plants in each irradiation condition. ^2^ Numbers in parenthesis indicates total number of rearrangement events/total number of plants in each irradiation condition. Data for dose 1 (~50% of *D*_q_) and dose 2(~75% of D_q_) in each irradiation condition were merged.

The spectrum of SBSs is shown in **Figure 4B**. The transition to transversion ratio (*T*_i_/*T*_v_) was in the range of 0.7 to 1.1 and no significant difference was observed between the irradiation treatments. The GC content of the TAIR10 reference sequence is 36%; therefore, the ratio of G:C pairs to A:T pairs (G:C/A:T) was 0.56 (36/64). However, the G:C/A:T ratio of the original nucleotides, which underwent base substitution, ranged between 0.7 and 1.8. These values were significantly higher than the value for TAIR10 except for dry-seed irradiation with carbon ions. This result indicated that the SBSs were prone to occur in G:C pairs rather than A:T pairs. This tendency was likely to be more evident with seedling irradiation than with dry-seed irradiation. The distribution of deletion sizes is shown in **Figure 4C**. The carbon ions resulted in a lower proportion of single-base deletions than gamma rays irrespective of the material.

**Figure 5:**
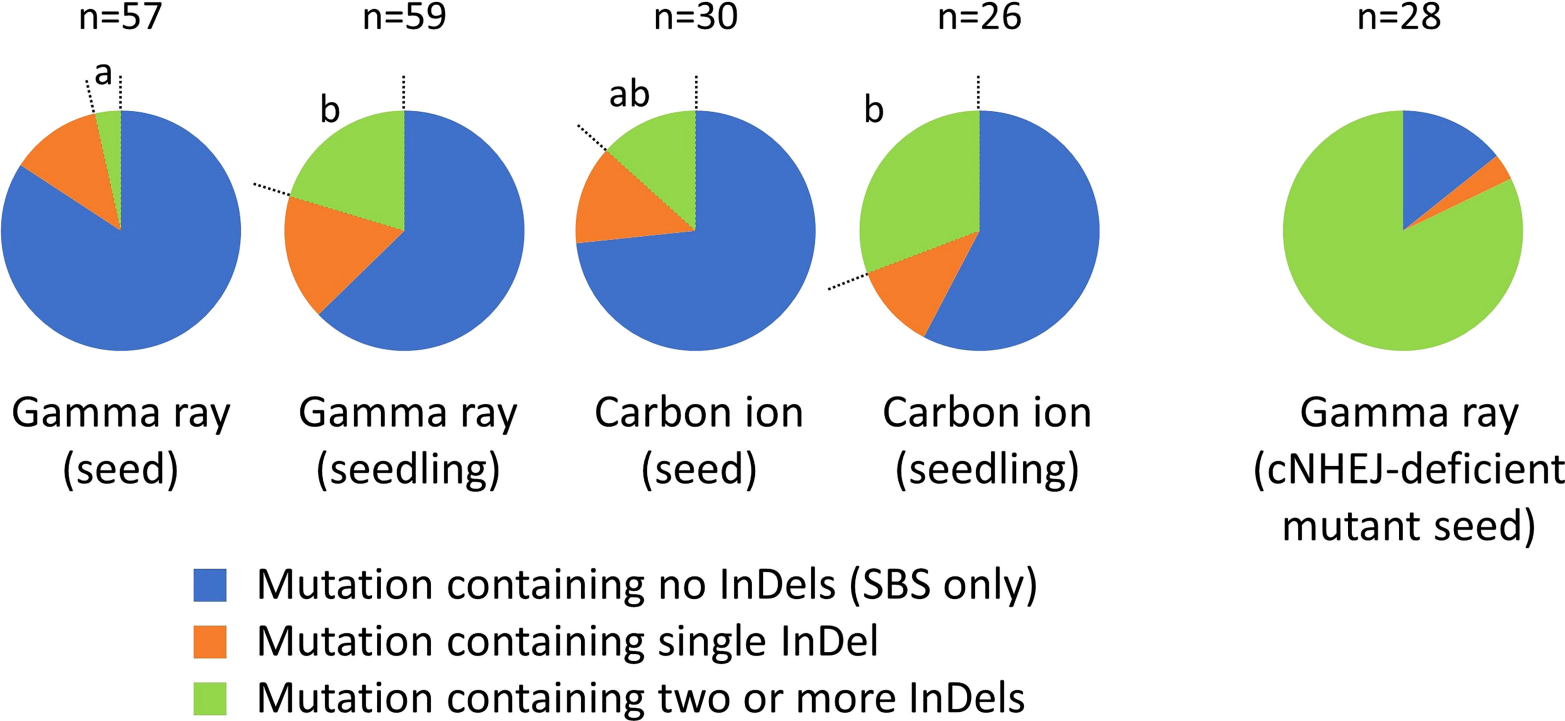
Complexity of the complex-type mutation. Different lowercase letters indicate significant differences in the proportion of complex-type mutations containing two or more InDels (Fisher’s exact test with multiple comparison correction, *p*< 0.05). Data for dose 1 (~50% of *D*_q_) and dose 2 (~75% of *D*_q_) in each irradiation treatment were merged. The data in this figure include both homozygous and heterozygous mutations. Data for *ku70* and *ligIV* mutants is from Du et al. (2020).

Complex-type mutations were subclassified into three groups, based on the number of InDels involved, to compare the complexity between the irradiation treatments (**Figure 5**). The data for doses 1 and 2 in each irradiation treatment were merged. Seedling irradiation with gamma rays and carbon ions both induced a significantly higher proportion of complex-type mutations containing two or more InDels, compared with dry-seed irradiation with gamma rays. This finding suggested that, at least in the case of gamma rays, seedling irradiation resulted in a higher frequency of more complicated complex-type mutations than dry-seed irradiation.

### 3.5 Number of protein-coding genes with non-synonymous mutations

**Figure 6:**
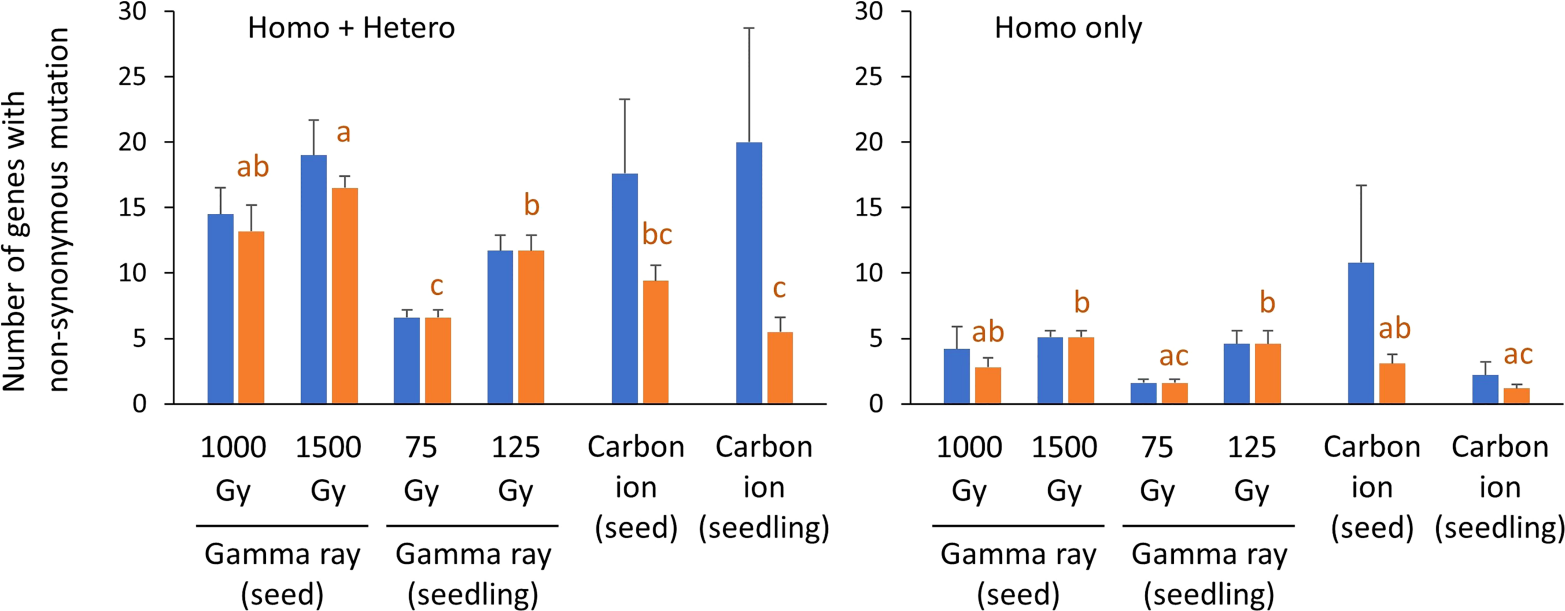
Number of protein-coding genes with non-synonymous mutations per sample. The blue bars indicate mean ± standard error in each irradiation condition. The orange bars indicate the same data but mutations that affect two more genes are excluded. The excluded deletions are listed in **Supplementary Table 5**. Transposable elements, pseudogenes, and non-coding RNA were not included. SVs with unknown overall structure were also not included. Data for dose 1 (~50% of *D_q_*) and dose 2 (~75% of *D_q_*) were merged for carbon ion irradiation. Different lowercase letters indicate a significant difference (one-way ANOVA with multiple comparison test, p< 0.05.

The number of protein-coding genes with non-synonymous mutations is shown in **Figure 6** and **Supplementary Table 4**. Data for doses 1 and 2 of carbon ion irradiation were merged because the MFs did not differ significantly and the mutation types were similar. Seedling irradiation with 75 or 125 Gy of gamma rays induced 1.6 and 4.6 non-synonymous homozygous mutations per plant on average, respectively (blue bars in **Figure 6**). These values were not significantly different from the values in the other irradiation treatments (one-way ANOVA with multiple comparison test, *p* = 0.27), probably because of the great inter-individual differences with carbon-ion irradiation. In addition, no significant difference was observed between the irradiation treatments in total number of mutations (homozygous + heterozygous, *p* = 0.48). The number of affected genes per plant varied considerably depending on the occurrence of a large deletion, particularly in the case of carbon-ion irradiation. Specifically, 60 and 32 genes were lost by 282 kb and 245 kb homozygous deletions induced by seed irradiation with carbon ions (**Supplementary Table 5**). If mutations that affected two or more genes were excluded, the frequency of non-synonymous homozygous mutations for seedling irradiation with 75 Gy gamma ray and seedling irradiation with carbon ions was significantly lower than for dry-seed irradiation with 1500 Gy gamma ray and seedling irradiation with 125 Gy gamma ray (orange bars in **Figure 6**). These differences corresponded well with the total mutation frequency (**Figure 3**).

## 4 Discussion

### 4.1 Combination of different algorithms is effective for detection of SVs

We demonstrated that the use of additional algorithms, namely Manta and Lumpy, enhanced the detection efficiency of SVs, particularly those in the heterozygous state (**Figure 2**, **Supplementary Table 3**). Under the present experimental conditions, Manta was more effective than Lumpy in detecting mutations that were not detected by our previous method using the GATK, Pindel, and BreakDancer algorithms. Li et al. (2019) reported that Manta was more effective than Pindel and BreakDancer for detecting SVs in rice. The homozygous:heterozygous ratio of the 30 SV events detected by our previous method was 12:18 (1:1.5). The homozygous:heterozygous ratio after adding the 20 newly detected SVs was 16:34 (1:2.1), which was close to Mendel’s theoretical ratio (1:2). Although some SVs may still be overlooked, the present results demonstrated that the combination of five algorithms greatly reduced the bias of detection efficiency depending on the types of mutation and zygosity. We succeeded in deducing the overall structure in 12 of the 20 (60%) newly detected SVs (**Supplementary Figure 1**, **Supplementary Table 3**). This highlights the difficulty of deducing the overall structure of heterozygous SVs. We consider that even short-read sequencing can detect most junctions of SV events; however, long-read sequencing technologies are indispensable to determine the overall structure of SV events, particularly in a heterozygous state and for SVs with three or more junctions.

### 4.2 Relationship between survival rate and MF may differ depending on the LET

At the equivalent doses for survival reduction, the MF was relatively higher with gamma irradiation than carbon-ion irradiation, and with dry-seed irradiation than seedling irradiation (**Figure 3**). Mutations induced by ionizing radiation have been extensively studied in Arabidopsis and rice. However, to date, few studies have compared ion-beam- and gamma-ray-induced mutations using the same material and method. Two independent studies have compared carbon-ion- and gamma-induced mutations in the M_4_–M_6_ generations of rice derived from irradiated dry seeds (Li et al., 2019; Yang et al., 2019). Gamma rays resulted in a higher MF than carbon ions in both studies and this is consistent with the present results. However, these two previous studies reported inconsistent results in that Yang et al. (2019) observed a higher proportion of InDel mutations with carbon-ion irradiation, whereas Li et al. (2019) observed no difference in InDel proportion between carbon-ion and gamma-ray irradiation. In addition, Li et al. (2019) reported that the frequency of SVs was higher with carbon-ion irradiation, whereas Yang et al. (2019) detected no SVs, although an inadequacy of the detection method was suggested by Zheng et al. (2021). We consider that further studies in rice using a greater number of plants, preferably in an earlier generation after irradiation, will be meaningful to resolve the above-mentioned discrepancy and for improved knowledge of mutagenesis, given that mutation techniques have been extensively applied in practical breeding of rice (Joint FAO/IAEA).

Interestingly, the MF at dose 2 (~75% *D*_q_) was higher than that at dose 1 (~50% *D*_q_) in the case of gamma rays, whereas no significant difference in MF was observed between the two doses in the case of carbon ions (**Figure 3**). This suggests that the relationship between the dose, as judged by the reduction in survival, and the MF in the M_2_ generation differed in the case of carbon ions and gamma rays. Recently, Lee et al. (2021) compared the phenotype-based MF (leaf color and morphological mutants) and the whole-genome sequencing (WGS)-based MF in Arabidopsis using a proton beam and gamma rays at three doses: near two-thirds of *D*_q_ (2/3 *D*_q_), *D*_q_, and 50% lethal dose (LD_50_). The phenotype-based MF in the M_2_ generation on a M_1_ plant basis showed the highest value at *D*_q_ in the case of protons, whereas the highest value was observed at LD_50_ in the case of gamma rays. The phenotype-based MF at 2/3 *D*_q_ was less than half that of the highest MF irrespective of irradiation source. In contrast, the WGS-based MF of the proton beam at *D*_q_ was comparable to the value at LD_50_ but was 1.2–1.3 times significantly higher than that of 2/3 *D*_q_. The WGS-based MF of gamma rays was examined only for *D*_q_ and was comparable to that of protons. Ichida et al. (2019) examined the chlorophyll MF and performed whole-exome sequencing (WES) in the M_2_ generation of rice derived from dry seeds irradiated with carbon ions. The chlorophyll MF peaked at 175 Gy, which was a slightly lower dose than the shoulder dose for seed survival (200 Gy), whereas the WES-based MF at 200 Gy was slightly higher than that at 150 Gy. These results suggest that a phenotype-based MF shows a peak at a dose slightly lower than *D*_q_ in the case of high-LET radiation, whereas the peak is observed at a dose slightly higher than *D*_q_ in the case of gamma rays. Furthermore, these studies and the present results suggest that, in the case of high-LET radiation, a WGS-based MF attains a plateau at a lower dose range relative to *D*_q_. This difference may reflect that high-LET radiation induces cell death more often in a meristem at a lower dose range relative to *D*_q_, owing to the greater severity of DNA damage compared with that induced by low-LET radiation. Consistent with this, we previously suggested that carbon ions induced a higher extent of cell death at ~50% *D*_q_ in Arabidopsis meristems compared with gamma rays by evaluating the size of the mutant sector generated by loss of heterozygosity (Hase et al., 2017). In practical mutation breeding, gamma rays may show the highest MF at a dose slightly higher than *D*_q_, whereas 50%–75% *D*_q_ may be a sufficiently high dose for high-LET radiation.

### 4.3 Carbon ions induced rearrangements more frequently than gamma rays in both dry-seed irradiation and seedling irradiation

Carbon ions induced rearrangements (SV and Del ≥ 100 bp) more frequently than gamma rays (**Figure 4A**, **Table 2**). This finding is consistent with results of previous studies showing that high-LET radiation induces more complex SVs with higher frequency than low-LET radiation after dry-seed irradiation (Hirano et al., 2015; Kazama et al., 2017). Importantly, the present results suggest that this tendency is observed with both dry-seed irradiation and seedling irradiation. Rearrangements are considered to be derived from rejoining of two distantly located DNA DSBs (Kazama et al., 2017; Li et al., 2019; Kitamura et al., 2022). Therefore, the present results suggested that carbon ions induced denser ionization and DNA breakage along the path of the ion particles that could result in the generation of rearrangements irrespective of the water content. The complexity and proximity of DNA damage has been shown to increase as the LET increases (Akamatsu et al., 2021), although the effect of water content remains unclear. In contrast, the difference in the ratio of Del (2–99 bp) between carbon ions and gamma rays observed with dry-seed irradiation was not evident with seedling irradiation (**Figure 4A**). This finding implies that, under the high water-content condition, the quality of individual DNA damage is less affected by the radiation quality. Taken together, these facts suggest that the frequency of rearrangement depends on the spatial distribution rather than the quality of DNA damage. Lee et al. (2021) reported that 100 MeV protons induced SVs more frequently than gamma rays at the respective *D*_q_ dose in Arabidopsis, although both radiation types induced a comparable total MF. These authors attributed the difference in frequency of SVs potentially to the difference in track structure, because protons reportedly induced more clustered DNA lesions compared with gamma rays (Calugaru et al., 2011). In addition, the finding that irradiation at *D*_q_ and LD_50_ showed a higher proportion of SVs than at 2/3 *D*_q_ was also consistent with the inference that the spatial distribution of DNA damage is associated with the generation of rearrangements.

### 4.4 SBS spectrum may vary with irradiation material

If the original nucleotide that underwent base-substitution is considered, the G:C/A:T ratio is 3.1 (75/24) (Ossowski et al., 2010) and 2.0 (47/24) (Hase et al., 2020) in the case of spontaneous mutation. This is reasonable because spontaneous mutation includes a higher proportion of G:C to A:T transitions. The SBS spectrum broadened by irradiation decreases the G:C/A:T ratio; however, the observed G:C/A:T ratios were still significantly higher than the ratio for the TAIR10 reference sequence, except for dry-seed irradiation with carbon ions (**Figure 4B**). Given that the reported spontaneous mutation frequencies (7 × 10^−9^/bp, Ossowski et al., 2010; 1 × 10^−8^/bp, Hase et al., 2020) are extremely low compared with the radiation-induced MF, the present results indicated that the G:C pair is more likely than the A:T pair to give rise to a SBS after irradiation. Ichida et al. (2019) reported that the G:C pair was 1.6 times more prone to mutate than the A:T pair for carbon-ion-induced SBS in rice detected by WES. Interestingly, it is likely that dry-seed irradiation showed a lower G:C/A:T ratio compared with seedling irradiation (**Figure 4B**). This may be associated with the relatively higher proportion of A:T to T:A transitions, which was commonly observed in dry-seed irradiation with gamma rays and carbon ions (yellow bars in **Figure 4B**). The relatively higher proportion of A:T to T:A transitions is often observed after dry-seed irradiation in Arabidopsis (Du et al., 2017; Kazama et al., 2017; Lee et al, 2021; Du et al., 2022; Kitamura et al., 2022), except in the study by Belfield et al. (2012). An Arabidopsis mutant that lacks UVH1, a nucleotide excision repair (NER)-associated endonuclease, spontaneously induced G:C to A:T transitions as well as A:T to T:A transitions at a one-digit higher frequency compared with the wild type (Willing et al., 2016). The contribution and accuracy of the NER pathway may be involved. If induction of weak/leaky alleles or gain-of-function alleles by ionizing radiation is expected, an even unbiased induction of all six possible SBS types is preferable, because it results in a wider variation of amino acid changes (Li et al., 2017). In this context, the presents results suggest that seedling irradiation with gamma rays is beneficial to induce less-biased nucleotide changes compared with the other material–radiation combinations (**Figure 4B**).

### 4.5 Seedling irradiation induced more complicated complex-type mutations

Subclassification of the complex-type mutations suggested that seedling irradiation induced more complicated complex-type mutations than dry-seed irradiation (**Figure 5**). This might be associated with the pathway for rejoining broken DNA ends. Canonical non-homologous end joining (cNHEJ) is considered to be the predominant pathway for DSB repair in higher eukaryotes (Shen et al., 2017). The cNHEJ pathway uses few or no homologies to rejoin the DSB ends and may cause small alterations at the junctions (Pannunzio et al., 2018). When the cNHEJ pathway is impaired or cannot complete the repair, backup pathways, such as alternative end joining and single-strand annealing, are considered to contribute to DSB repair. Compared with the cNHEJ pathway, the backup pathways are non-conservative because they use longer homologies for rejoining after extensive resectioning of the DSB ends. Complex-type mutations containing two or more InDels are more often observed after gamma-ray irradiation of Arabidopsis mutant seeds that lack *Ku70* or *Ligase IV*, the major components of the cNHEJ pathway (**Figure 5**; Du et al., 2020). Similarly, frequent induction of longer deletions is observed in cNHEJ-deficient Arabidopsis and rice after DNA cleavage with site-directed artificial nucleases (Osakabe et al., 2010; Qi et al., 2013; Nishizawa-Yokoi et al., 2016; Shen et al., 2017; Schmidt et al., 2019). The present results suggest that, in the case of seedling irradiation, backup pathways may contribute slightly more frequently to DSB repair than in the case of dry-seed irradiation.

## Data availability statement

The whole-genome sequencing data of seedling irradiation with gamma rays analyzed in this study were deposited in the DNA Data Bank of Japan Sequence Read Archive (https://ddbj.nig.ac.jp/dra) with the accession number DRA015438. The sequencing data of the other irradiation combinations have been registered with the accession numbers DRA006578 and DRA008787.

## Author contributions

YH designed and performed the experiment. KS analyzed the raw sequence data. YH and SK interpreted the data. YH wrote the first draft of the manuscript. All authors read and approved the final manuscript. All authors contributed to the article and approved the submitted version.
