# Supplementary material for: Comparative analysis of seed and seedling irradiation with gamma rays and carbon ions for mutation induction in Arabidopsis
Source: Front Plant Sci. 2023 Apr 6;14:1149083. doi: 10.3389/fpls.2023.1149083 (PMC10117944; doi:10.3389/fpls.2023.1149083)
Supplement: Supplementary file 1 [file DataSheet_1.pdf]

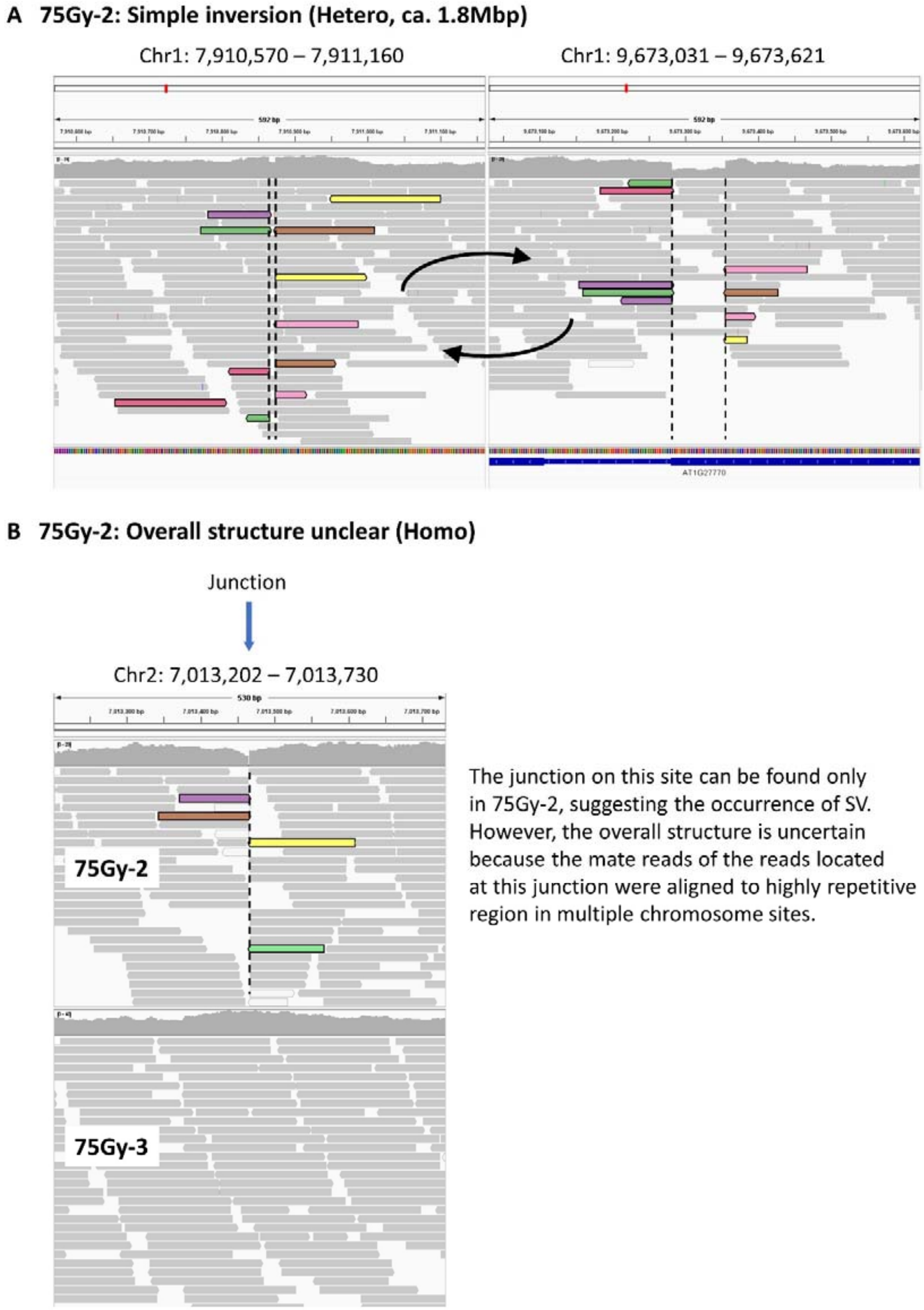
A 75Gy-2: Simple inversion (Hetero, ca. 1.8Mbp)
Chr1: 7,910,570 – 7,911,160
Chr1: 9,673,031 – 9,673,621
592 bp
AT1G27770
B 75Gy-2: Overall structure unclear (Homo)
Junction
Chr2: 7,013,202 – 7,013,730
530 bp
75Gy-2
75Gy-3
The junction on this site can be found only in 75Gy-2, suggesting the occurrence of SV. However, the overall structure is uncertain because the mate reads of the reads located at this junction were aligned to highly repetitive region in multiple chromosome sites.

C 75Gy-3: Simple inversion (Hetero, ca. 3.6 Mbp)

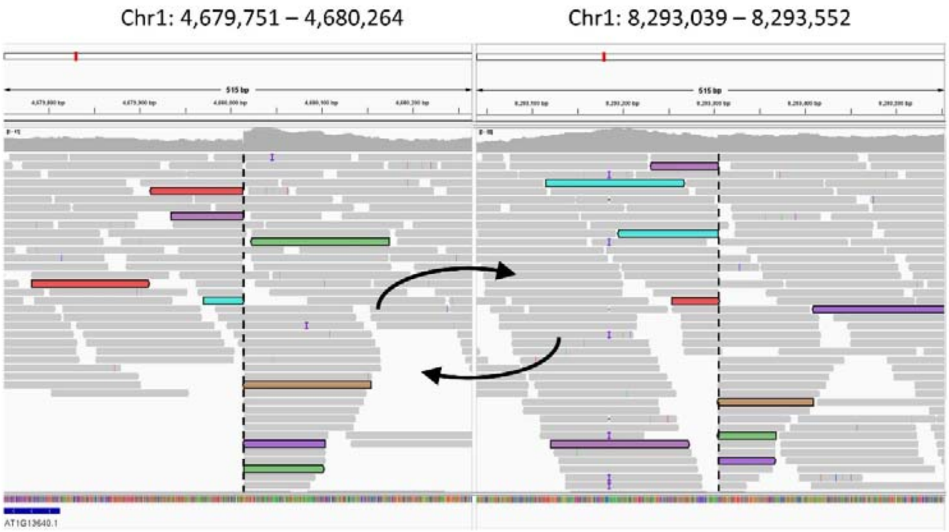

D 125Gy-2: Overall structure unclear (Hetero)

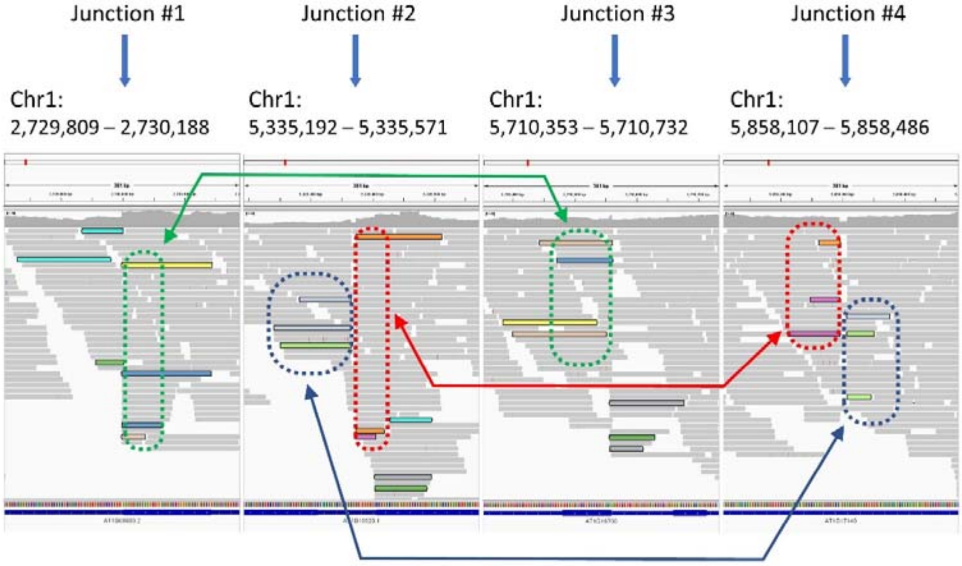

This SV includes at least four heterozygous junction points on chromosome 1. According to the mate pair information, the right side of junction #1 is connected with the left side of junction #3. The left side of junction #2 is connected with the right side of junction #4. The connection between the right side of junction #2 and the left side of junction #4 is also suggested. However, the relationship among the left side of junction #1, the right side of #2 and the right side of #3 is uncertain. These facts suggests the complex translocation, inversion and duplication, however, the overall structure is unclear.

E 125Gy-4: Overall structure unclear (Heteo)

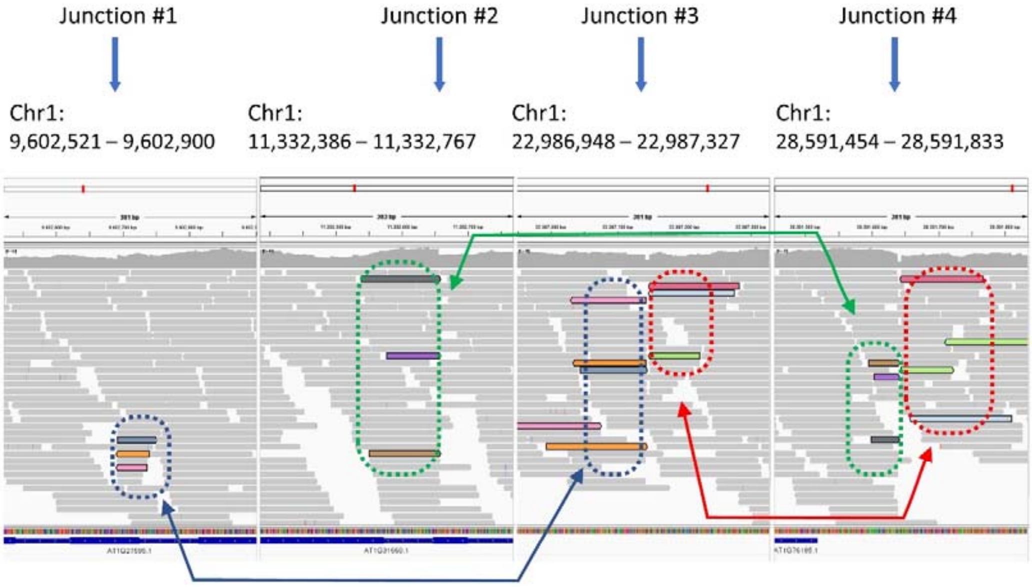

This SV includes at least four heterozygous junction points on chromosome 1. According to the mate pair information, the left side of junction #2 is connected with the left side of junction #4. The right side of junction #3 is connected with the right side of junction #4. In addition, the connection between the junction #1 and the left side of junction #3 is suggested, however, the mutual relationship is uncertain.

F 125Gy-4: Simple inversion (Heteo, ca. 6.6 Mbp)

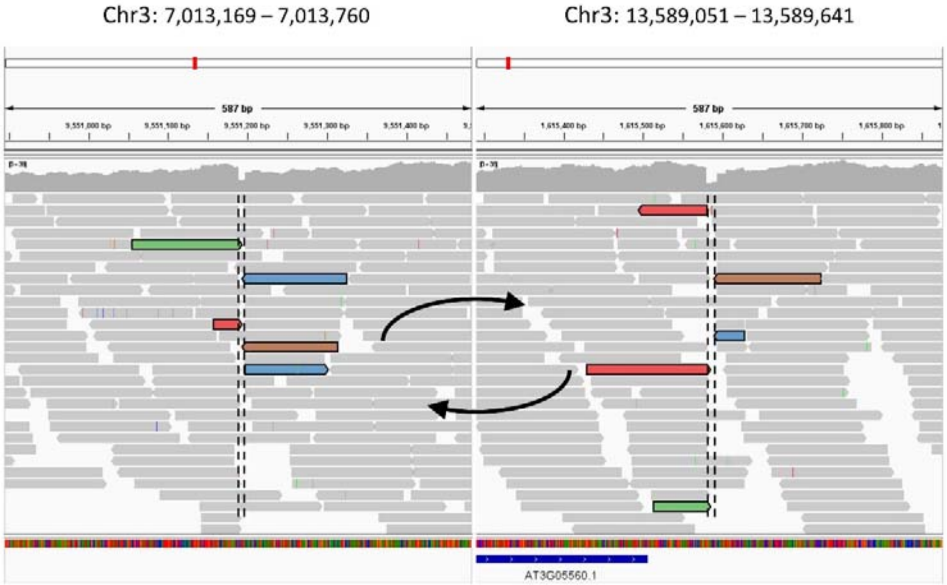

Supplementary Figure 1. Captured screen image of structural variations detected by the Manta and Lumpy algorithms in seedling irradiation with gamma rays. Each pair of sequencing reads are shown in the same color.

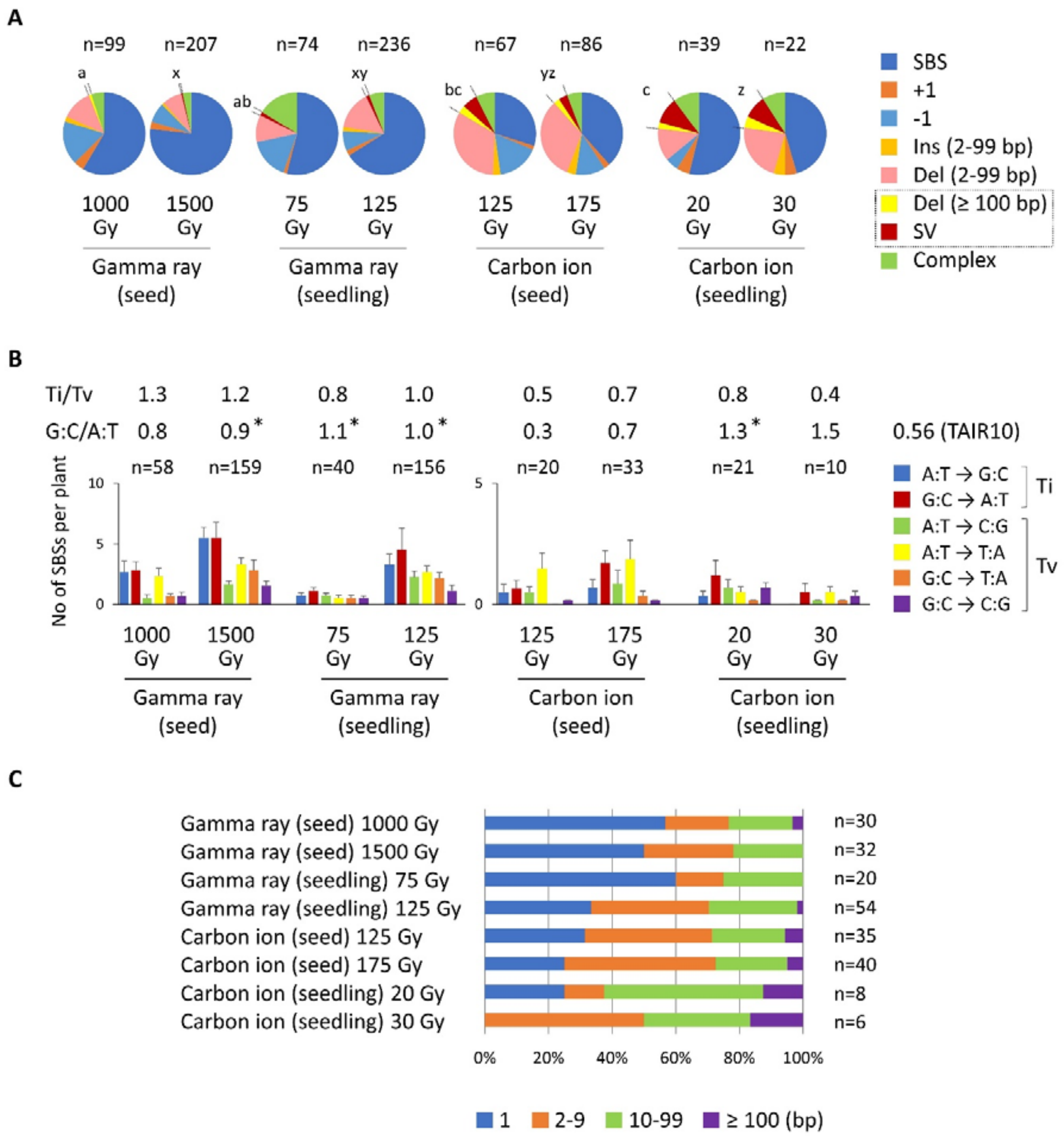

Supplementary Figure 2. Characterization of mutations induced in Arabidopsis dry seeds and 7-day-old seedlings irradiated with 17.3 MeV/u carbon ions or gamma rays. The data in this figure include only homozygous mutations. (A) All mutation types detected in each treatment. The proportions of rearrangements (Deletion ≥ 100 bp and structural variation [SV]) are indicated by dotted lines. Statistical comparisons of the proportions of rearrangements were performed at the equivalent dose on survival reduction. Different lowercase letters indicate a significant difference (Fisher's exact test with multiple comparison correction, $p < 0.05$). (B) Spectra of single-base substitutions. Complementary substitutions (e.g., G:C to A:T and C:G to T:A) were merged. The T_i/T_v ratio represents the ratio of total transition to transversion events. The G:C/A:T ratio represents the ratio of G:C pairs to A:T pairs in the original nucleotides that underwent substitutions. Asterisks indicate significant differences from the A:T/G:C ratio (0.56) of the TAIR10 reference sequence (Fisher's exact test, $p < 0.05$). (C) Distribution of deletion sizes.

Supplementary Table 1. The number of mutation sites during the filtering process in 20 M_2 plants derived from seedling irradiation.

The raw number of mutation sites on chromosome 1 to 5 called by GATK Haplotype Caller	11,483
Unique homozygous (Genotype =1/1) or heterozygous (0/1) mutations among twenty samples	1,350
Unique mutation with allele frequency > 25% and the allele frequency < 5% for all the other nineteen samples	1,047 *
Number of reliable mutations after confirmation with Integrative Genomics Viewer	819 ** (Homo 306, Hetero 513)

* Most of 58 complex-type mutations (more than two consecutive SBSs, or more than two SBSs and/or short InDels identified with a gap of less than 10 bases) detected by GATK was independently counted at this step. Therefore, the actual number at this step is around 990.

** 28 out of 819 mutations were also detected by Pindel and/or BreakDancer algorithms.

Supplementary Table 2. Summary of mapping results of *Arabidopsis* M_2 plants derived from seven-day-old seedlings irradiated with gamma rays.

sample	Total mapped base (Mb)	Mean depth of coverage	Coverage above 10x (%)	Coverage above 25x (%)
125Gy-1	5,274	44.1	99.9	83.6
125Gy-2	5,629	47.1	99.9	89.1
125Gy-3	5,056	42.3	99.8	77.0
125Gy-4	4,449	37.2	99.6	55.6
125Gy-5	5,453	45.6	99.9	90.9
125Gy-6	5,727	47.9	99.9	90.9
125Gy-7	5,416	45.3	99.9	88.2
125Gy-8	4,929	41.3	99.8	70.8
125Gy-9	5,050	42.3	99.8	66.5
125Gy-10	4,477	37.5	99.2	43.3
75Gy-1	4,979	41.7	99.8	74.7
75Gy-2	4,336	36.3	99.5	47.5
75Gy-3	4,518	37.8	99.8	69.8
75Gy-4	5,207	43.6	99.9	80.8
75Gy-5	5,134	43.0	99.9	84.1
75Gy-6	4,402	36.8	99.7	59.1
75Gy-7	4,909	41.1	99.8	75.2
75Gy-8	5,022	42.0	99.8	78.4
75Gy-9	4,300	36.0	99.5	48.1
75Gy-10	4,775	40.0	99.7	62.8
Mean	4,952	41.4	99.8	71.8
SD	433	3.6	0.2	15.0

Supplementary Table 3. Mutation events detected by the Manta and Lumpy algorithms.

Sample	Type of mutation	Zygosity	Algorithm*	Deduced alteration
Gamma ray × dry seeds				
1000-2-1	Deletion	Homo	M, L	Deletion (Chr.3, ca. 25 kb)
1000-6-1	SV	Hetero	M only	Reciprocal translocation (Chr.3 & 5)
1000-6-1	SV	Hetero	M only	Simple inversion (Chr.4, ca. 143 kb)
1500-1-1	SV	Hetero	M, L	Simple inversion (Chr.3, ca. 25 kb)
1500-7-1	SV	Hetero	M, L	Simple inversion (Chr.2, ca. 582 kb)
1500-5-1	Deletion	Hetero	M only	Deletion (Chr.1, ca. 86 kb)
Gamma ray × seedlings				
75Gy-2	SV	Hetero	M, L	Simple inversion (Chr.1, ca. 1.8 Mb)
	SV	Homo	M only	Overall structure unclear (Chr.2) [1]
75Gy-3	SV	Hetero	M, L	Simple inversion (Chr.1, ca. 3.6 Mb)
125Gy-2	SV	Hetero	M, L	Overall structure unclear (Chr.1) [2]
125Gy-4	SV	Hetero	M only	Overall structure unclear (Chr.1) [3]
	SV	Hetero	L only	Simple inversion (Chr.3, ca. 6.6 Mb)
125Gy-5	SV	Homo	M, L	Reciprocal translocation (Chr.1 & 2)
	SV	Hetero	M, L	Overall structure unclear (Chr.1& 2) [4]
125Gy-8	SV	Hetero	M, L	Overall structure unclear (Chr.2) [5]
125Gy-9	SV	Hetero	M, L	Reciprocal translocation (Chr.1 & 2)
	Complex	Hetero	M only	Deletion (49 bp) and SBS
Carbon ion × dry seeds				
125-2-2	SV	Homo	M, L	Simple inversion (Chr.5, ca. 356 kb)
125-4-1	SV	Homo	M, L	Simple inversion (Chr.3, ca. 926 kb)
125-12-1	SV	Hetero	M only	Overall structure unclear (Chr.5) [6]
175-6-1	SV	Hetero	M, L	Overall structure unclear (Chr.2) [7]
Carbon ion × seedlings				
20-3-1	Deletion	Hetero	M only	Deletion (Chr.1, ca. 256 kb)
30-3-4	Deletion	Hetero	M only	Deletion (Chr.2, ca. 380 kb)
30-4-1	SV	Hetero	M only	Reciprocal translocation (Chr.2 & 5)
20-5-3	SV	Hetero	M, L	Overall structure unclear (Chr.5) [8]

* M; Manta, L; Lumpy.

[1] There is one homozygous junction point on chromosome 2, but the sequence reads at this junction points connected with highly repetitive region. Screen capture images of IGV are shown in Supplementary Figure 1B.

[2] This SV includes four major heterozygous junction points on chromosome 1. Screen capture images of IGV are shown in Supplementary Figure 1D.

[3] This SV includes at least four major heterozygous junction points on chromosome 1. Screen capture images of IGV are shown in Supplementary Figure 1E.
[4] This SV includes at least four major heterozygous junction points on chromosome 1 and 2.
[5] This SV includes ~4.1 Mbp inversion and duplication on chromosome 2.
[6] This SV includes at least two major heterozygous junction points on chromosome 5, but the sequence reads at this junction points connected with highly repetitive region.
[7] This SV includes at least four heterozygous junctions on chromosome 2.
[8] This SV includes at least three heterozygous junctions on chromosome 5.

Supplementary Table 4. Number of protein-coding genes with non-synonymous mutation per plant.

		Homo	Hetero	Total
Gamma ray (seed)	1000 Gy	4.2 ± 1.7<br>(2.8 ± 0.7)	10.3 ± 1.8	14.5 ± 2.7<br>(13.2 ± 2.0)
	1500 Gy	5.1 ± 0.5	13.9 ± 3.0<br>(11.4 ± 1.2)	19.0 ± 2.7<br>(16.5 ± 0.9)
Gamma ray (seedling)	75 Gy	1.6 ± 0.3	5.0 ± 0.5	6.6 ± 0.6
	125 Gy	4.6 ± 1.0	7.1 ± 1.3	11.7 ± 1.2
Carbon (seed)	125 and 175 Gy	10.8 ± 5.9<br>(3.1 ± 0.7)	6.8 ± 1.1<br>(6.3 ± 1.0)	17.6 ± 5.7<br>(9.4 ± 1.2)
Carbon (seedling)	20 and 30 Gy	2.2 ± 1.0<br>(1.2 ± 0.3)	17.8 ± 8.7<br>(4.3 ± 1.1)	20.0 ± 8.7<br>(5.5 ± 1.1)

Values are mean ± standard error in each irradiation condition. Numbers in parentheses are the values when mutations that affect two more genes are excluded. Transposable elements, pseudogenes and non-coding RNA were not included. SVs with unknown overall structure were also not included. Data for dose 1 (~50% of *Dq*) and dose 2 (~75% of *Dq*) were merged for carbon ion irradiation.

Supplementary Table 5. Deletions larger than 5 kb.

Sample	Mutation	Zygosity	Number of affected protein- coding genes
Gamma ray × Seeds 1000 Gy 2-1	25.6-kb deletion	Homo	8
Gamma ray × Seeds 1500 Gy 5-1	86.3-kb deletion	Hetero	20
Carbon × Seeds 125 Gy 12-1	6.3-kb deletion	Hetero	6
	245.4-kb deletion	Homo	32
Carbon × Seeds 175 Gy 4-1	281.9-kb deletion	Homo	60
Carbon × Seedlings 20 Gy 3-1	256.8-kb deletion	Hetero	76
Carbon × Seedlings 20 Gy 4-4	25.6-kb deletion*	Homo	12
Carbon × Seedlings 30 Gy 4-1	380.4-kb deletion	Hetero	86

* This deletion occurred at the junction of 431-kb inversion.
